# Author Correction: Annual cycle observations of aerosols capable of ice formation in central Arctic clouds

**DOI:** 10.1038/s41467-022-33777-w

**Published:** 2022-10-12

**Authors:** Jessie M. Creamean, Kevin Barry, Thomas C. J. Hill, Carson Hume, Paul J. DeMott, Matthew D. Shupe, Sandro Dahlke, Sascha Willmes, Julia Schmale, Ivo Beck, Clara J. M. Hoppe, Allison Fong, Emelia Chamberlain, Jeff Bowman, Randall Scharien, Ola Persson

**Affiliations:** 1 Department of Atmospheric Science, Colorado State University, Fort Collins, CO USA; 2 Cooperative Institute for Research in Environmental Sciences, University of Colorado, Boulder, CO USA; 3 Physical Sciences Laboratory, National Oceanic and Atmospheric Administration, Boulder, CO USA; 4 Climate Sciences Division, Alfred-Wegener-Institut, Helmholtz-Zentrum für Polar- und Meeresforschung, Potsdam, Germany; 5 Environmental System Analysis and Modeling, Universität Trier, Trier, Germany; 6 Extreme Environments Research Laboratory, École Polytechnique Fédérale de Lausanne, Lausanne, Switzerland; 7 Biosciences Division, Alfred-Wegener-Institut, Helmholtz-Zentrum für Polar- und Meeresforschung, Bremerhaven, Germany; 8 Scripps Institution of Oceanography, University of California, San Diego, CA USA; 9 Department of Geography, University of Victoria, Victoria, BC Canada

**Keywords:** Environmental sciences, Climate sciences

**Correction to**: *Nature Communications* 10.1038/s41467-022-31182-x, published online 20 June 2022

The original version of this Article contained an error in Fig. 4, in which the y-axis should read “INPs (L^−1^ of air)” instead of “INPs x 10^−3^ (L^−1^ of air)”. This has been corrected in both the PDF and HTML versions of the Article.

