# Supplementary material for: Annual cycle observations of aerosols capable of ice formation in central Arctic clouds
Source: Nat Commun. 2022 Jun 20;13:3537. doi: 10.1038/s41467-022-31182-x (PMC9209516; doi:10.1038/s41467-022-31182-x)
Supplement: Supplementary file 1 — Supplementary Information [file 41467_2022_31182_MOESM1_ESM.pdf]

Supporting figures

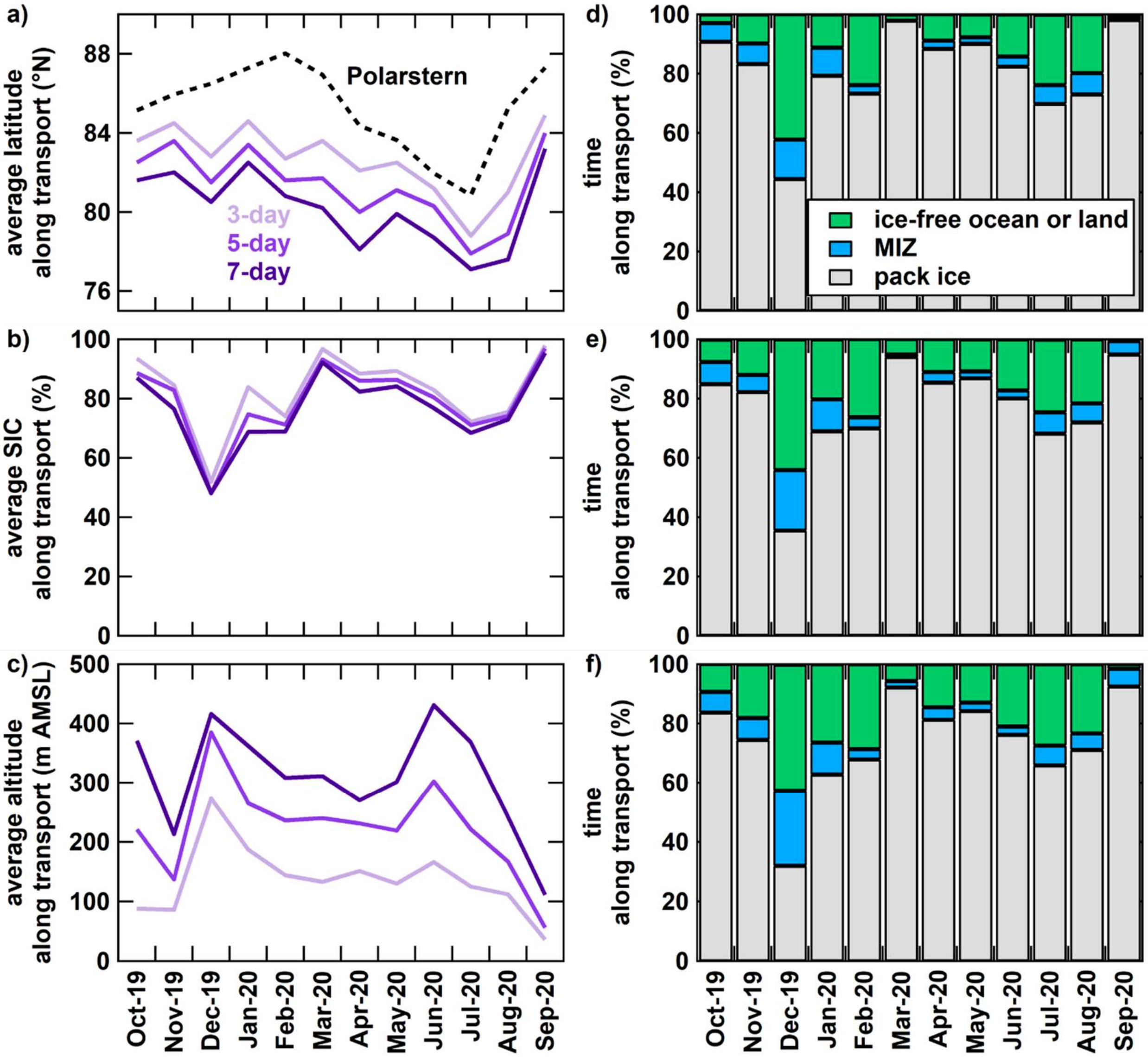

Supplementary Fig. 1. Comparison of trajectory parameters for 3, 5, and 7 days back in time ending at 30 m above mean sea level (m AMSL) above Polarstern. Parameters were averaged per month for all endpoints along trajectories ending at 00:00 universal coordinated time (UTC) and include a) average latitude, b) average sea ice concentration (SIC), and c) average altitude (in meters above mean sea level; m AMSL) within a 0.1-degree grid box around trajectory endpoints. Additionally, the land cover types along trajectory pathways were calculated for d) 3-day, e) 5-day, and f) 7-day backward trajectories using daily SIC data for pack ice (≥ 85% SIC), the marginal ice zone (MIZ; 15 – 85% SIC), and ice-free ocean or land (< 15% SIC). Polarstern's average location is also shown in panel a), for reference.

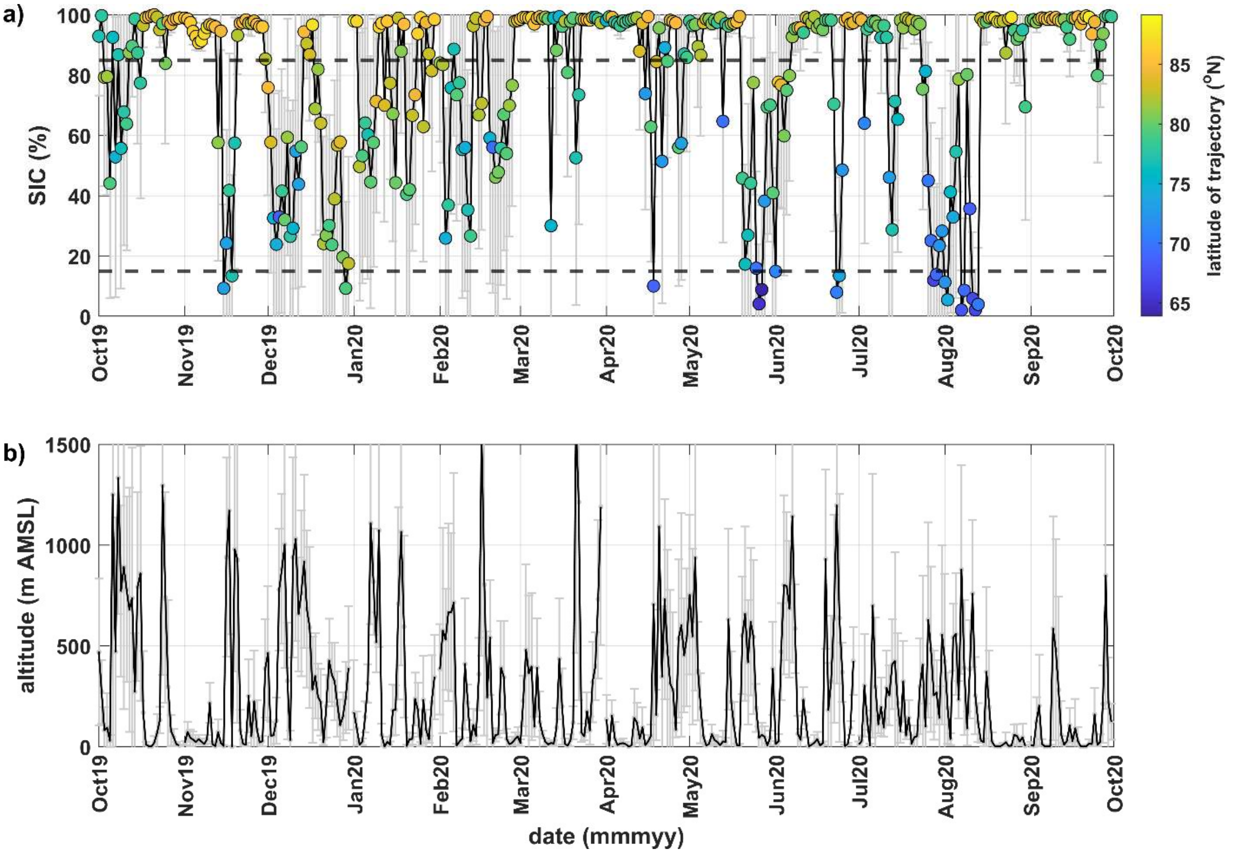

Supplementary Fig. 2. Spatial properties of airmass transport pathways to Polarstern. a) The average sea ice concentration (SIC) along each 5-day airmass backward trajectory endpoint. Trajectories were initiated daily at 00:00 universal coordinated time (UTC) and ending at 30 m above mean sea level (AMSL) above Polarstern with endpoints every 6 hours back in time. The dashed lines indicate the thresholds for pack ice (85–100%), the marginal ice zone (15–85%), and ice-free ocean or land (0–15%). Threshold definitions were obtained from the Norwegian Polar Institute (https://www.npolar.no/en/themes/the-marginal-ice-zone/). b) Corresponding altitude along each 5-day trajectory averaged at all end points. Error bars indicated standard deviation of the daily averages.

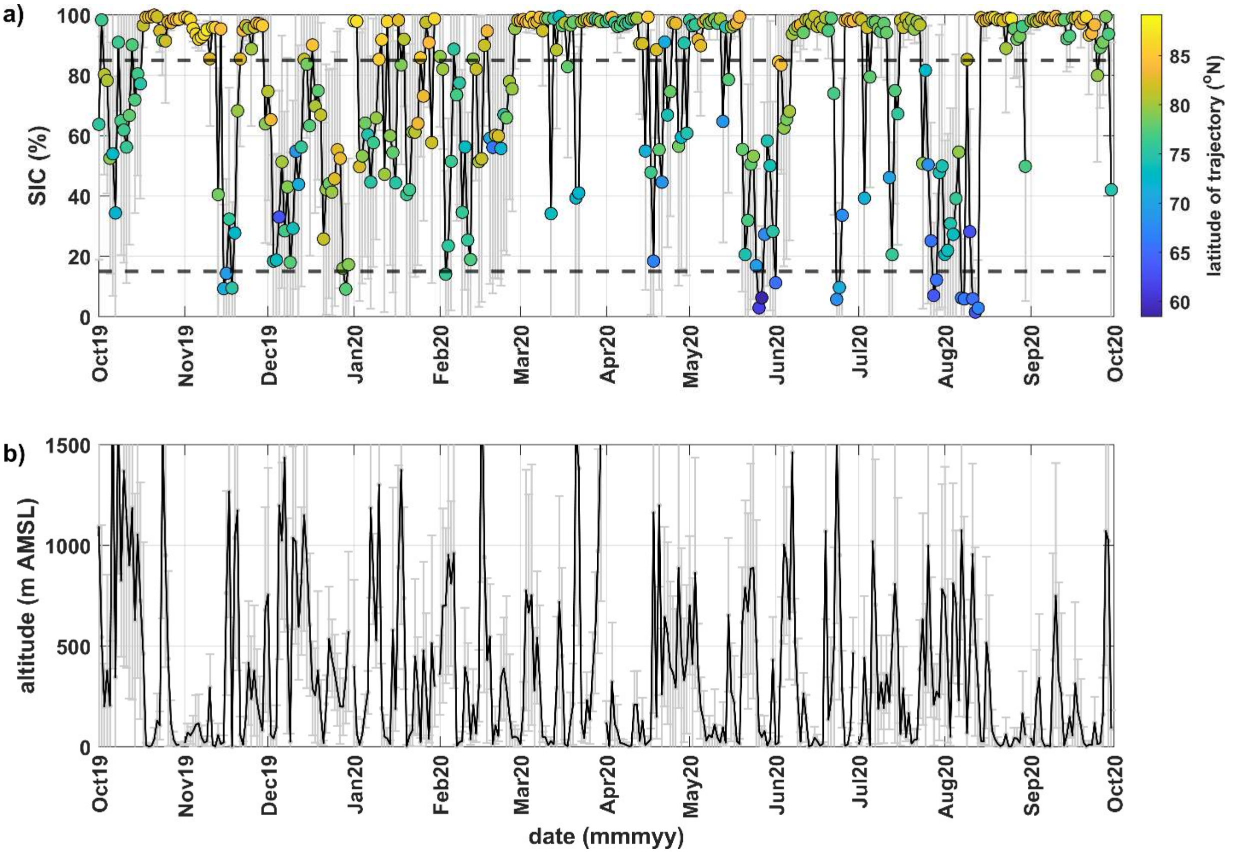

Supplementary Fig. 3. Spatial properties of airmass transport pathways to Polarstern. a) The average sea ice concentration (SIC) along each 7-day airmass backward trajectory endpoint. Trajectories were initiated daily at 00:00 universal coordinated time (UTC) and ending at 30 m above mean sea level (AMSL) above Polarstern with endpoints every 6 hours back in time. The dashed lines indicate the thresholds for pack ice (85–100%), the marginal ice zone (15–85%), and ice-free ocean or land (0–15%). Threshold definitions were obtained from the Norwegian Polar Institute (https://www.npolar.no/en/themes/the-marginal-ice-zone/). b) Corresponding altitude along each 7-day trajectory averaged at all end points. Error bars indicated standard deviation of the daily averages.

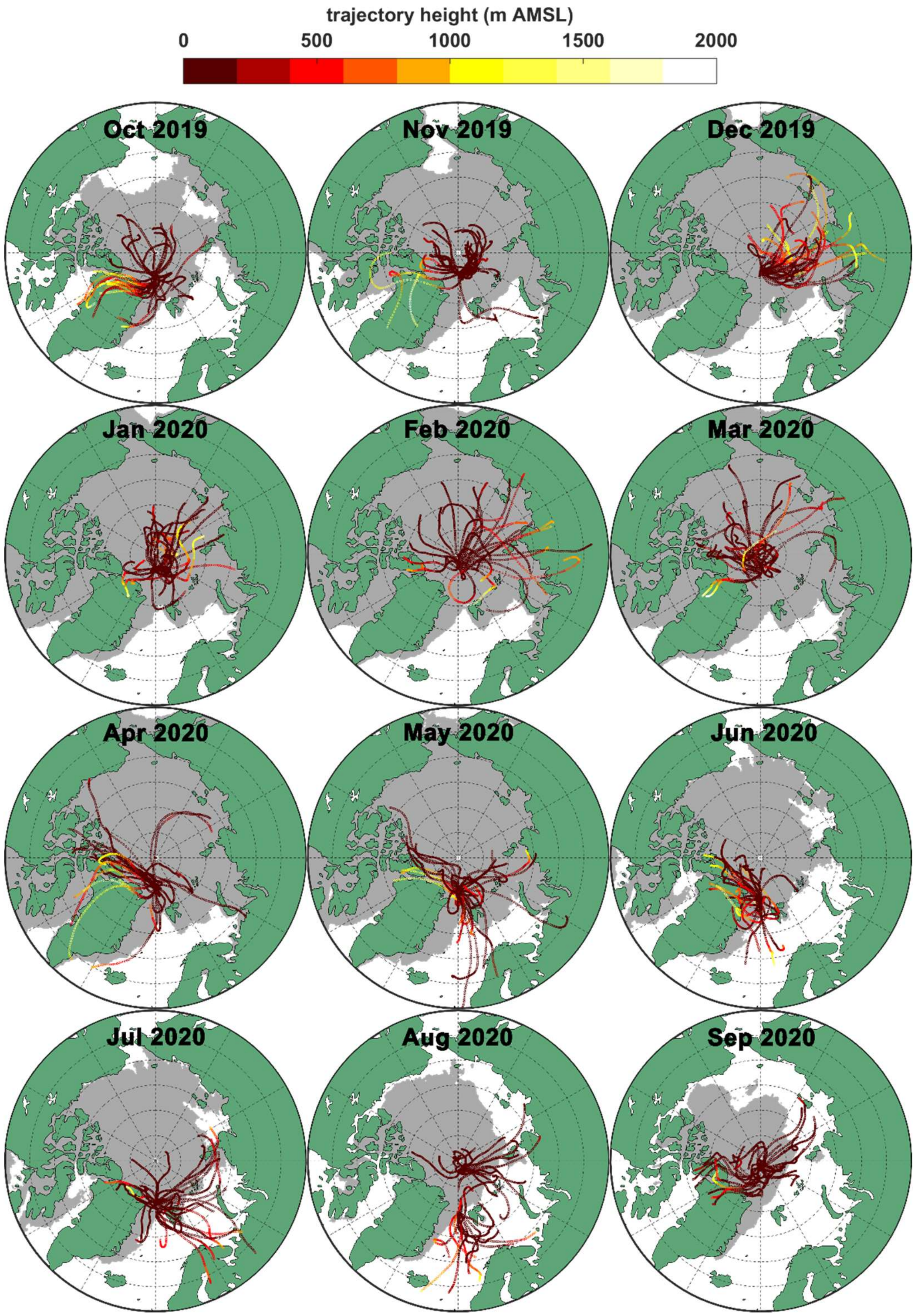

Supplementary Fig. 4. Monthly air mass transport pathways overlain monthly pan-Arctic sea ice concentration (SIC). Backward trajectories (3-day) from 00:00 universal coordinated time (UTC) and ending at 30 m above mean sea level (AMSL) above Polarstern are colored by trajectory height (in m above mean sea level; m AMSL) shown per month. Only months are shown that contained ice nucleating particle (INP) data that spanned the entire period. White coloring corresponds to open water. Maps were created by the authors using MathWorks MATLAB (https://www.mathworks.com/products/matlab.html).

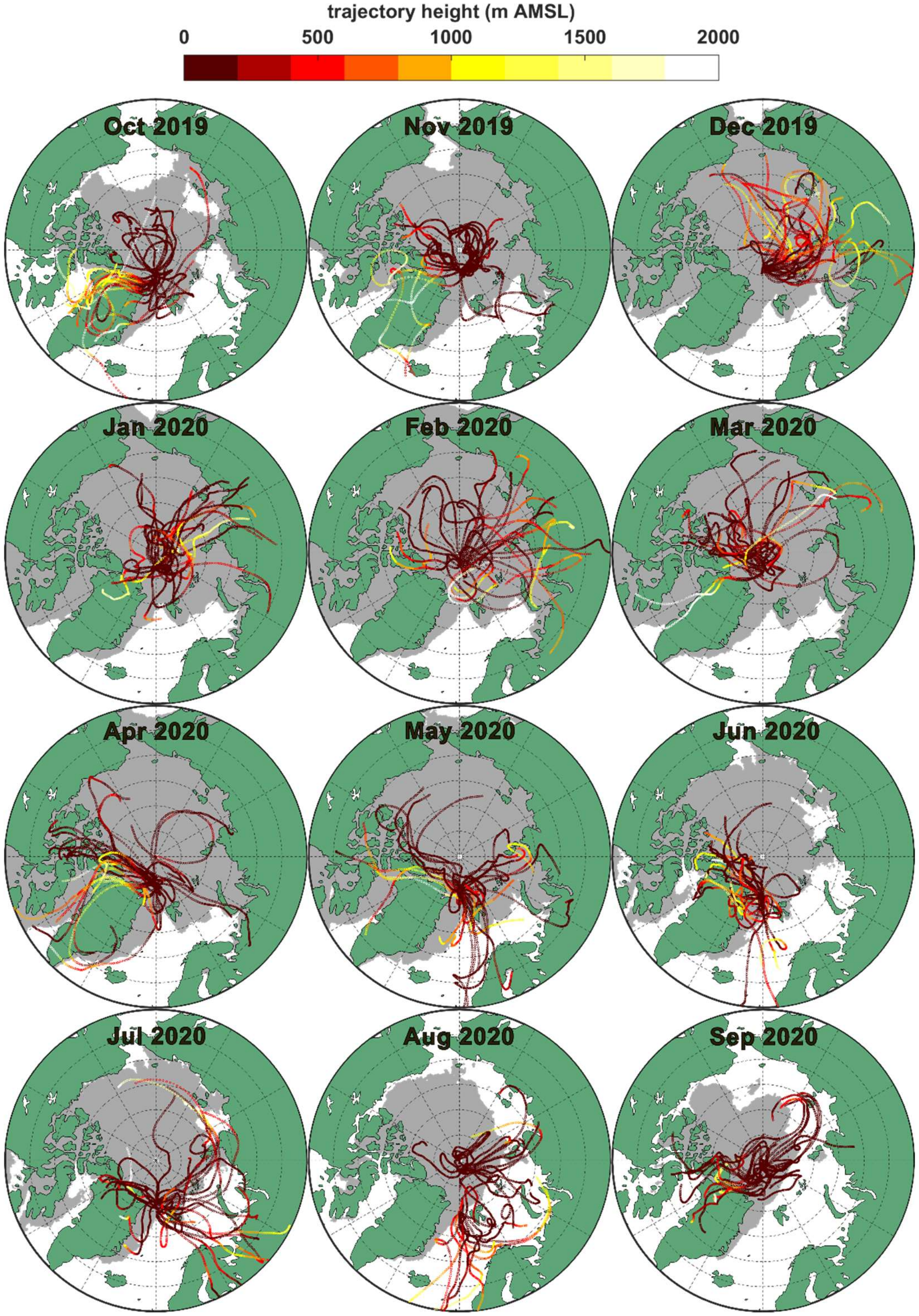

Supplementary Fig. 5. Monthly air mass transport pathways overlain monthly pan-Arctic sea ice concentration (SIC). Backward trajectories (5-day) from 00:00 universal coordinated time (UTC) and ending at 30 m above mean sea level (AMSL) above Polarstern are colored by trajectory height (in m above mean sea level; m AMSL) shown per month. Only months are shown that contained ice nucleating particle (INP) data that spanned the entire period. White coloring corresponds to open water. Maps were created by the authors using MathWorks MATLAB (https://www.mathworks.com/products/matlab.html).

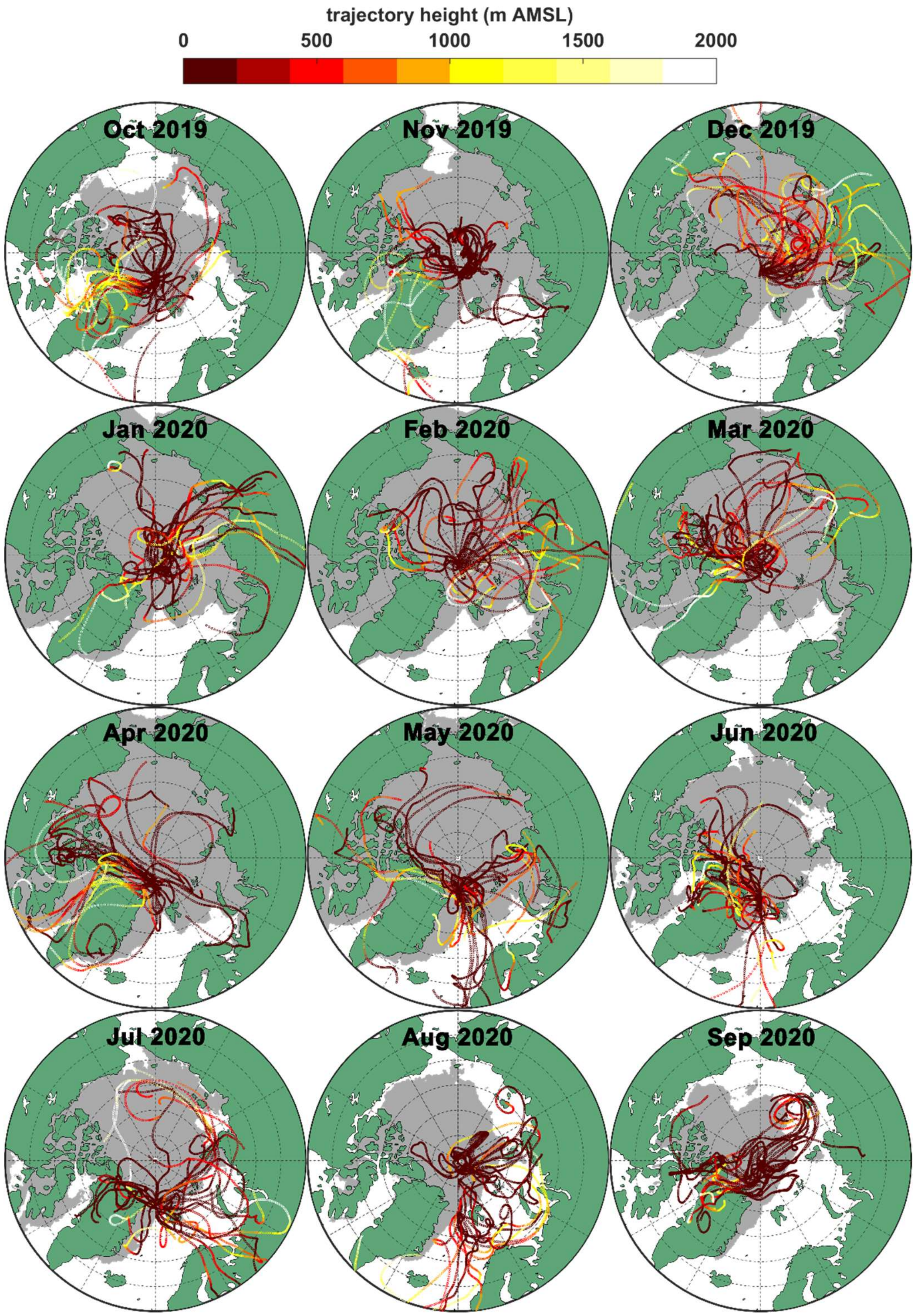

Supplementary Fig. 6. Monthly air mass transport pathways overlain monthly pan-Arctic sea ice concentration (SIC). Backward trajectories (7-day) from 00:00 universal coordinated time (UTC) and ending at 30 m above mean sea level (AMSL) above Polarstern are colored by trajectory height (in m above mean sea level; m AMSL) shown per month. Only months are shown that contained ice nucleating particle (INP) data that spanned the entire period. White coloring corresponds to open water. Maps were created by the authors using MathWorks MATLAB (https://www.mathworks.com/products/matlab.html).

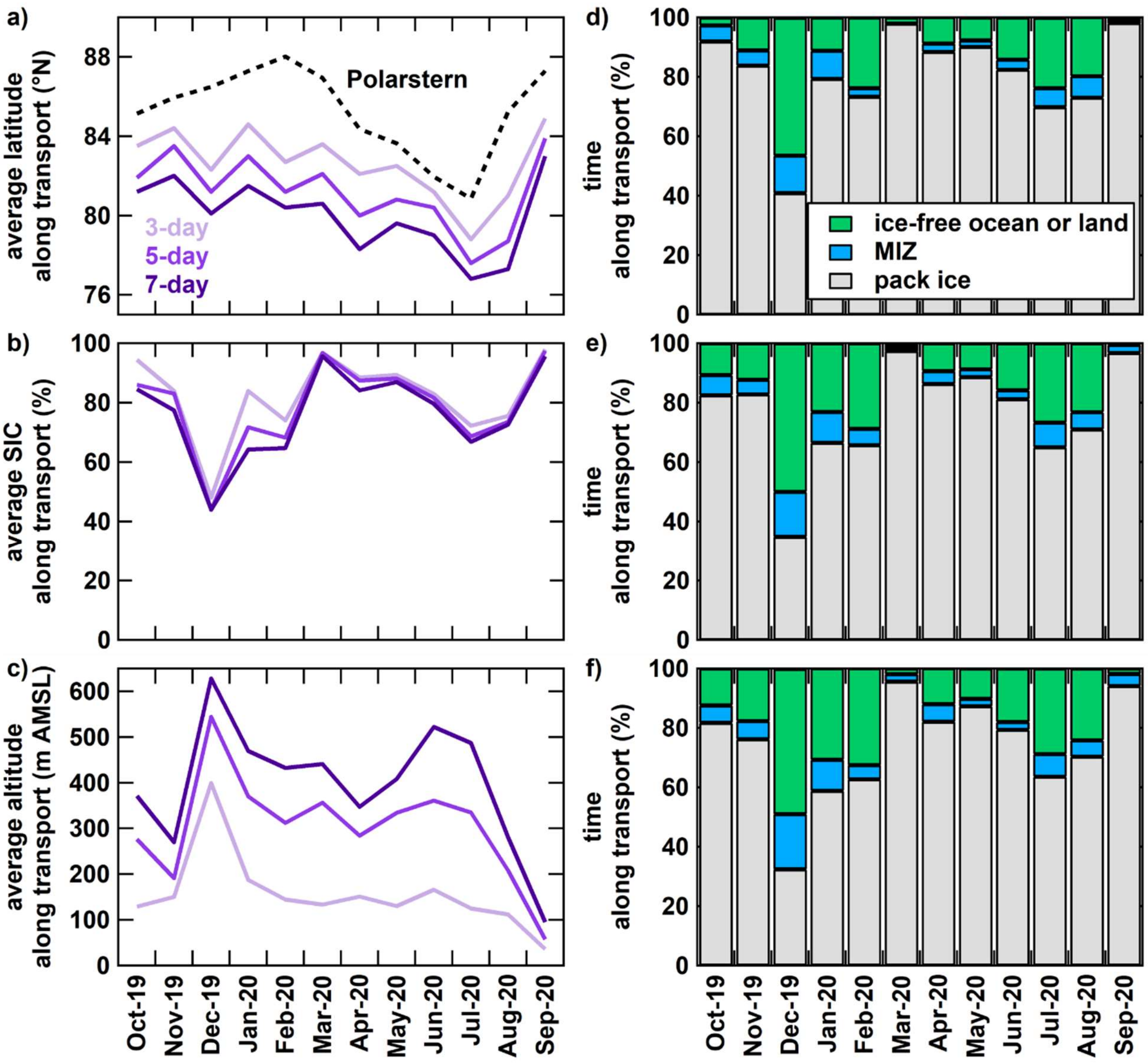

Supplementary Fig. 7. Comparison of trajectory parameters for 3, 5, and 7 days back in time ending at 100 m above mean sea level (m AMSL) above Polarstern. Parameters were averaged per month for all endpoints along trajectories ending at 00:00 universal coordinated time (UTC) and include a) average latitude, b) average sea ice concentration (SIC), and c) average altitude (in meters above mean sea level; m AMSL) within a 0.1-degree grid box around trajectory endpoints. Additionally, the land cover types along trajectory pathways were calculated for d) 3-day, e) 5-day, and f) 7-day backward trajectories using daily SIC data for pack ice (≥ 85% SIC), the marginal ice zone (MIZ; 15 – 85% SIC), and ice-free ocean or land (< 15% SIC). Polarstern's average location is also shown in panel a), for reference.

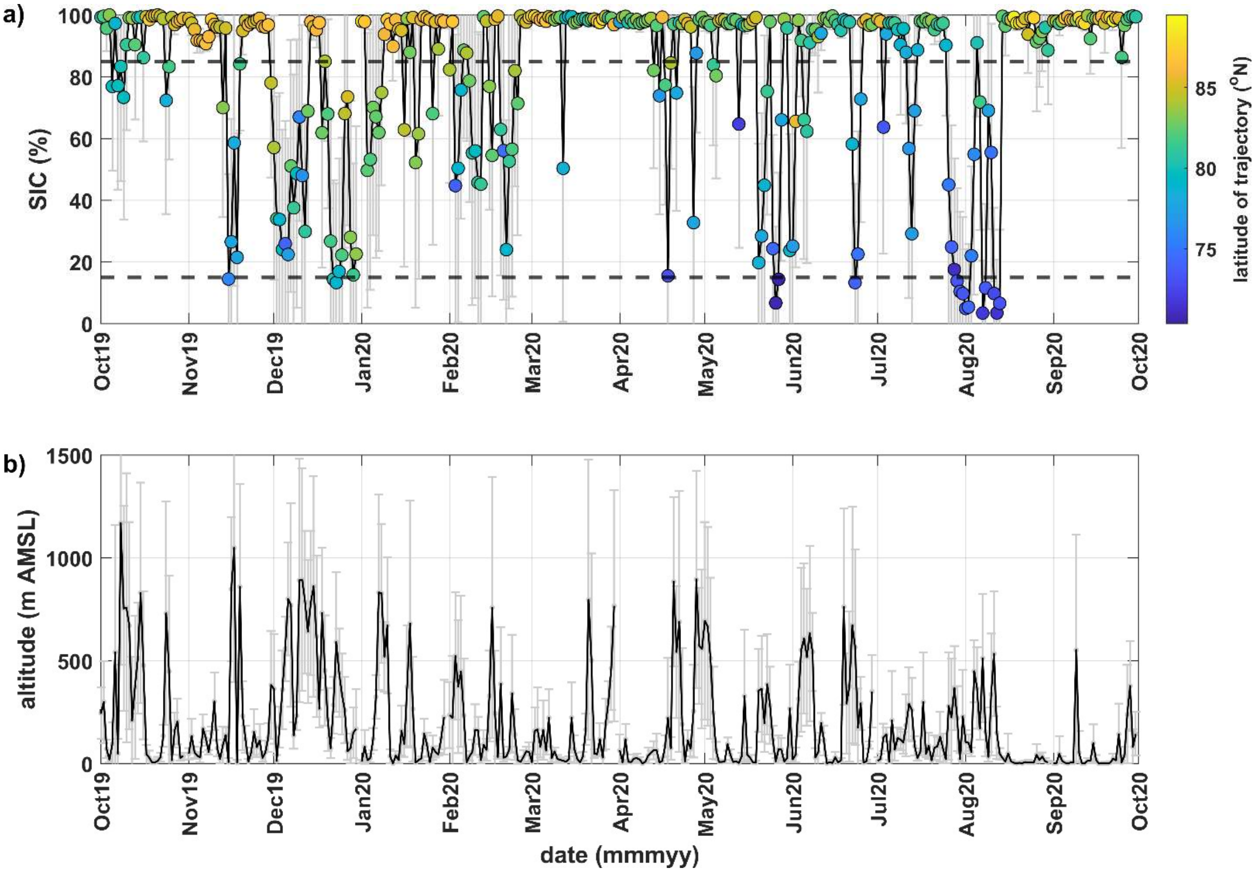

Supplementary Fig. 8. Spatial properties of airmass transport pathways to Polarstern. a) The average sea ice concentration (SIC) along each 3-day airmass backward trajectory endpoint. Trajectories were initiated daily at 00:00 universal coordinated time (UTC) and ending at 100 m above mean sea level (AMSL) above Polarstern with endpoints every 6 hours back in time. The dashed lines indicate the thresholds for pack ice (85–100%), the marginal ice zone (15–85%), and ice-free ocean or land (0–15%). Threshold definitions were obtained from the Norwegian Polar Institute (https://www.npolar.no/en/themes/the-marginal-ice-zone/). b) Corresponding altitude along each 3-day trajectory averaged at all end points. Error bars indicated standard deviation of the daily averages.

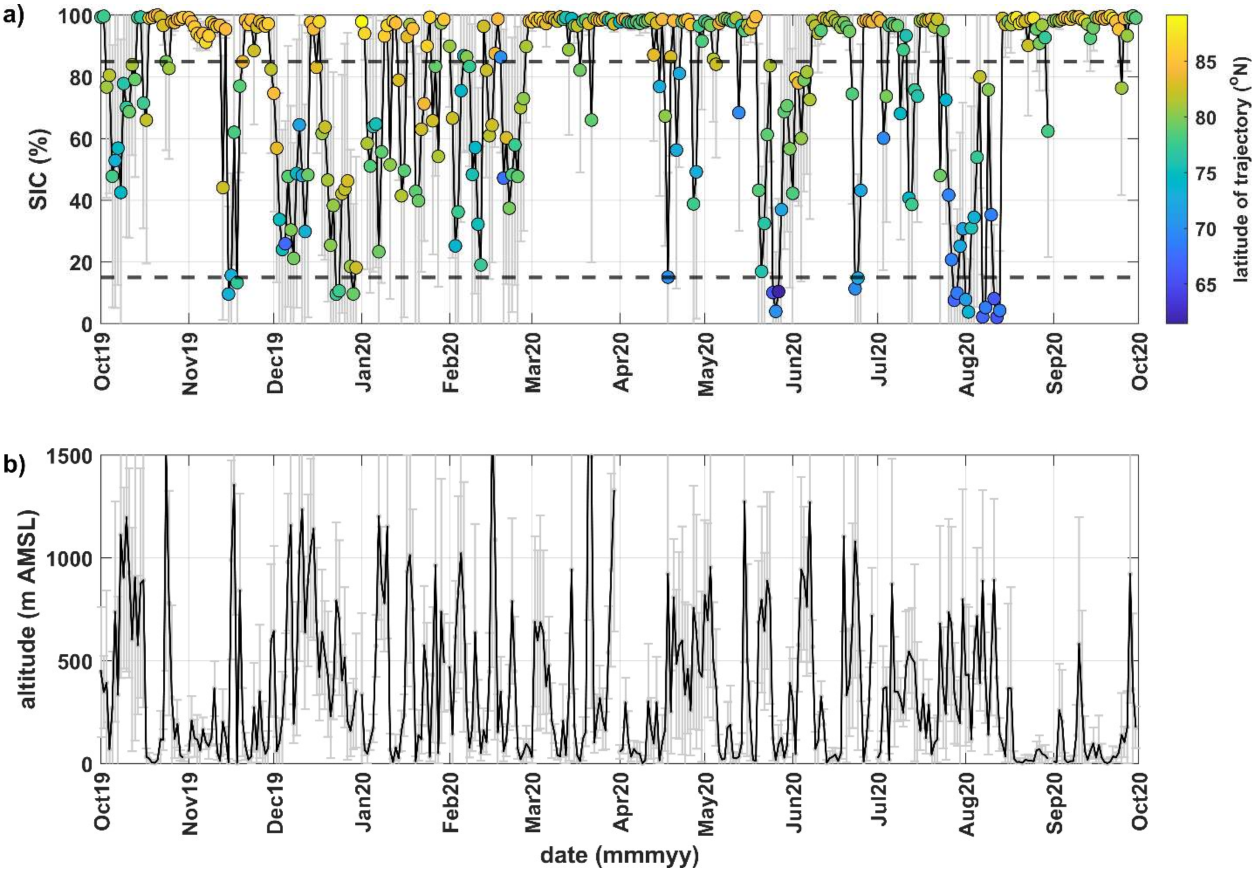

Supplementary Fig. 9. Spatial properties of airmass transport pathways to Polarstern. a) The average sea ice concentration (SIC) along each 5-day airmass backward trajectory endpoint. Trajectories were initiated daily at 00:00 universal coordinated time (UTC) and ending at 100 m above mean sea level (AMSL) above Polarstern with endpoints every 6 hours back in time. The dashed lines indicate the thresholds for pack ice (85–100%), the marginal ice zone (15–85%), and ice-free ocean or land (0–15%). Threshold definitions were obtained from the Norwegian Polar Institute (https://www.npolar.no/en/themes/the-marginal-ice-zone/). b) Corresponding altitude along each 5-day trajectory averaged at all end points. Error bars indicated standard deviation of the daily averages.

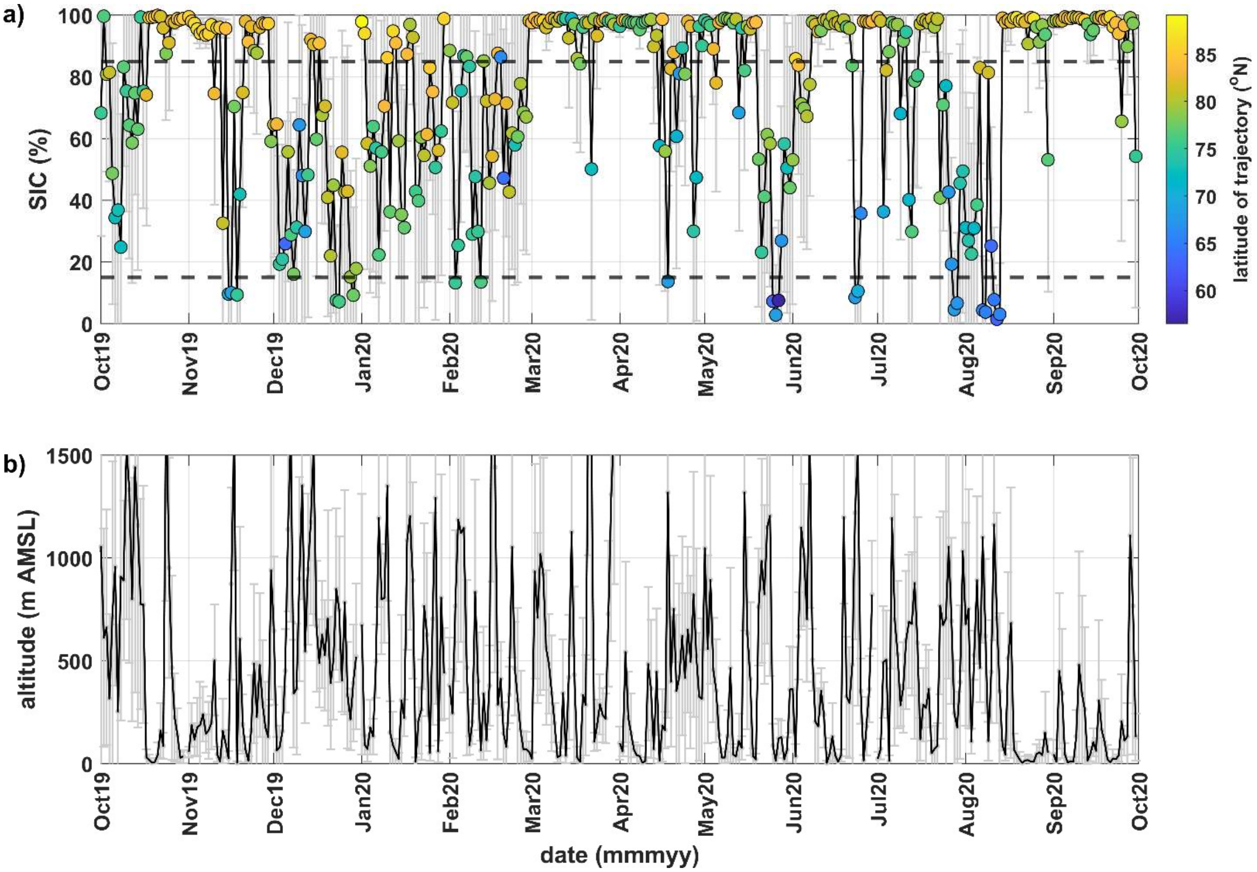

Supplementary Fig. 10. Spatial properties of airmass transport pathways to Polarstern. a) The average sea ice concentration (SIC) along each 7-day airmass backward trajectory endpoint. Trajectories were initiated daily at 00:00 universal coordinated time (UTC) and ending at 100 m above mean sea level (AMSL) above Polarstern with endpoints every 6 hours back in time. The dashed lines indicate the thresholds for pack ice (85–100%), the marginal ice zone (15–85%), and ice-free ocean or land (0–15%). Threshold definitions were obtained from the Norwegian Polar Institute (https://www.npolar.no/en/themes/the-marginal-ice-zone/). b) Corresponding altitude along each 7-day trajectory averaged at all end points. Error bars indicated standard deviation of the daily averages.

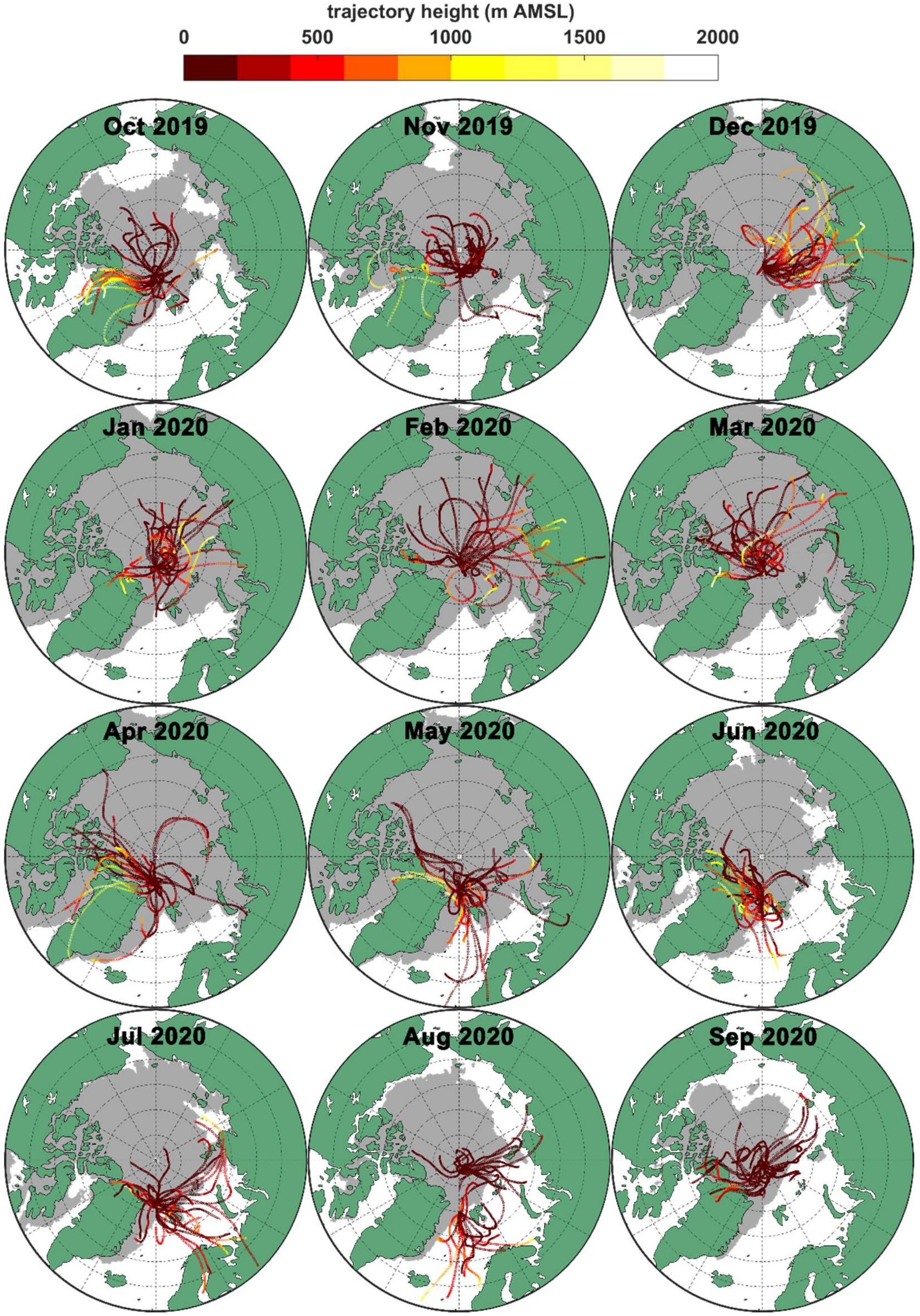

Supplementary Fig. 11. Monthly air mass transport pathways overlain monthly pan-Arctic sea ice concentration (SIC). Backward trajectories (3-day) from 00:00 universal coordinated time (UTC) and ending at 100 m above mean sea level (AMSL) above Polarstern are colored by trajectory height (in m above mean sea level; m AMSL) shown per month. Only months are shown that contained ice nucleating particle (INP) data that spanned the entire period. White coloring corresponds to open water. Maps were created by the authors using MathWorks MATLAB (https://www.mathworks.com/products/matlab.html).

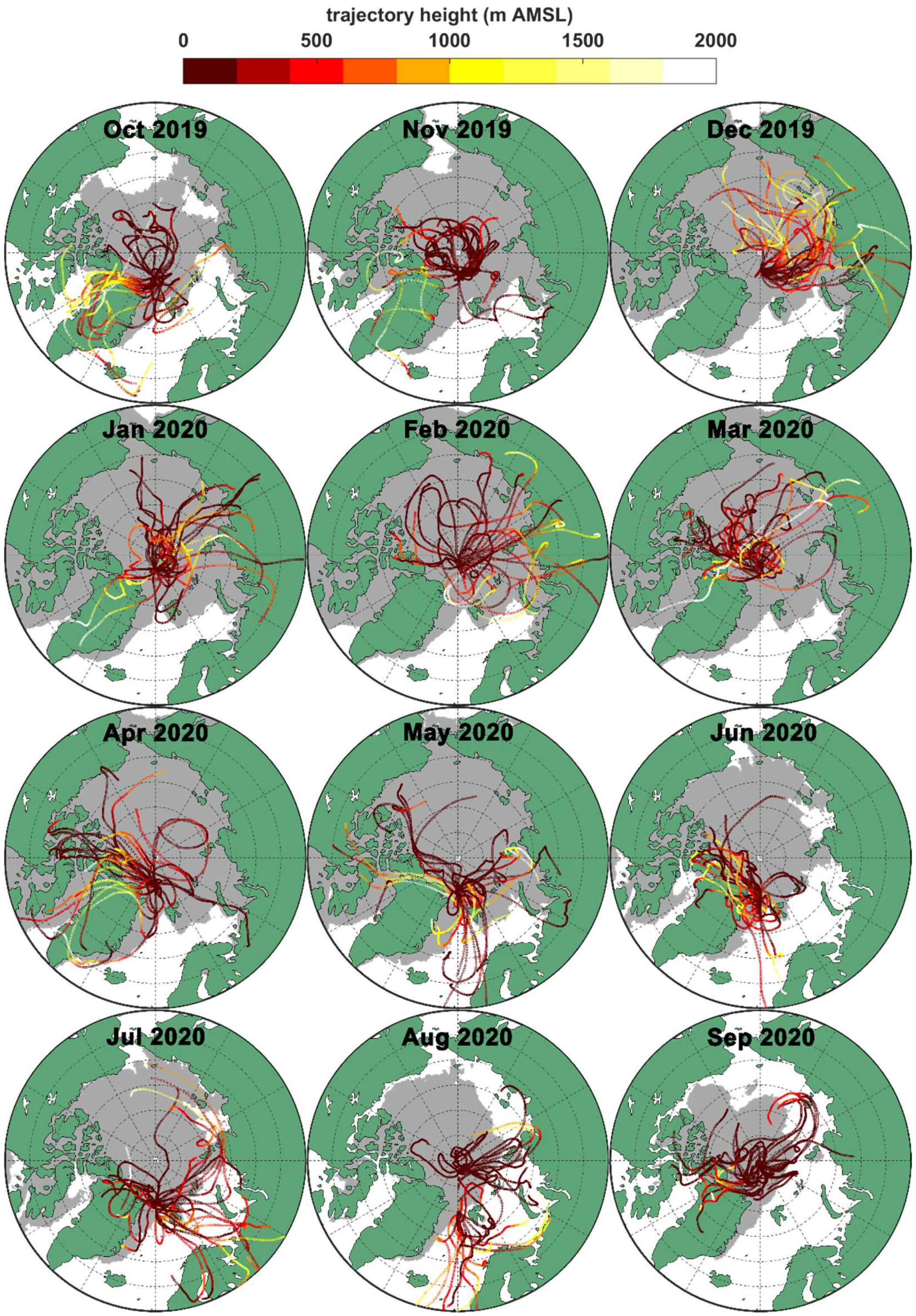

Supplementary Fig. 12. Monthly air mass transport pathways overlain monthly pan-Arctic sea ice concentration (SIC). Backward trajectories (5-day) from 00:00 universal coordinated time (UTC) and ending at 100 m above mean sea level (AMSL) above Polarstern are colored by trajectory height (in m above mean sea level; m AMSL) shown per month. Only months are shown that contained ice nucleating particle (INP) data that spanned the entire period. White coloring corresponds to open water. Maps were created by the authors using MathWorks MATLAB (https://www.mathworks.com/products/matlab.html).

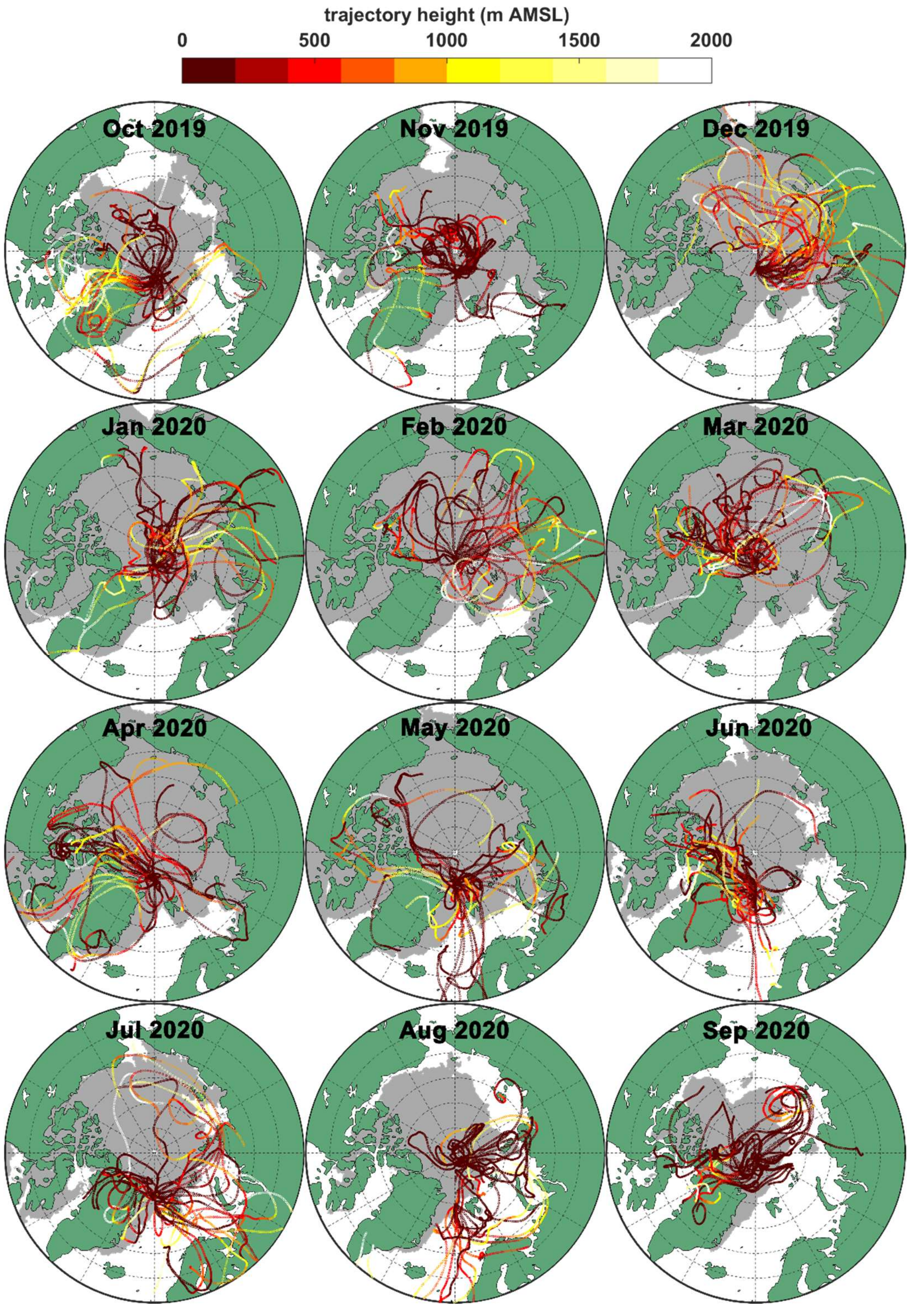

Supplementary Fig. 13. Monthly air mass transport pathways overlain monthly pan-Arctic sea ice concentration (SIC). Backward trajectories (7-day) from 00:00 universal coordinated time (UTC) and ending at 100 m above mean sea level (AMSL) above Polarstern are colored by trajectory height (in m above mean sea level; m AMSL) shown per month. Only months are shown that contained ice nucleating particle (INP) data that spanned the entire period. White coloring corresponds to open water. Maps were created by the authors using MathWorks MATLAB (https://www.mathworks.com/products/matlab.html).

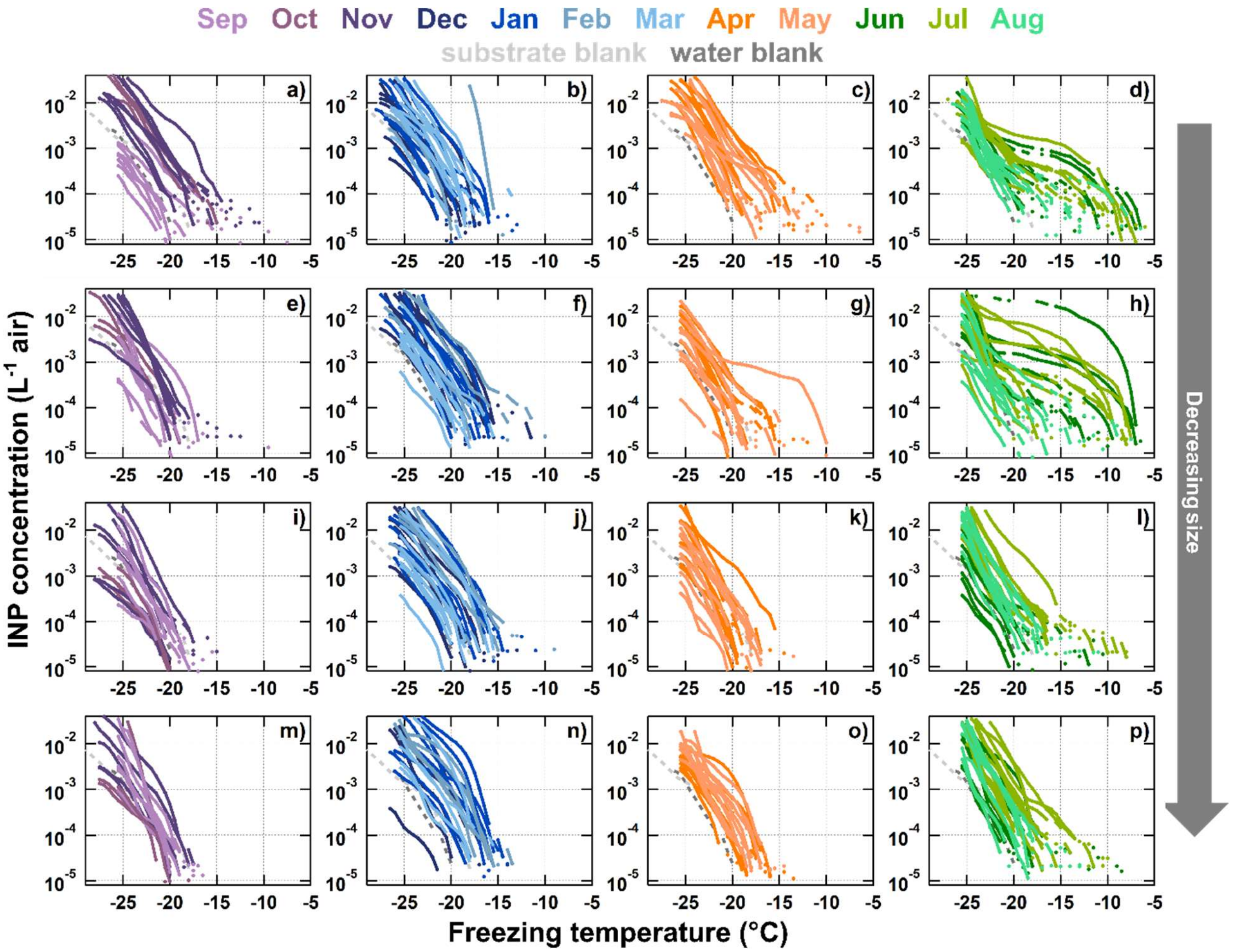

Supplementary Fig. 14. Seasonal cycle of size-resolved ice nucleating particles (INPs). Cumulative INP spectra are grouped by season to highlight the fall freeze-up (September–November), winter (December–March), spring transition (April–May), and summer melt (July–August) seasons (from left to right). Spectra are further sorted by aerosol diameter for a)–d) 3.0–12 µm, e)–h) 1.21–3.0 µm, i)–l) 340 nm–1.21 µm, and m)–p) 150–340 nm (from top to bottom). Dashed grey lines show the DRUM substrate in deionized water and plain deionized water blank controls.

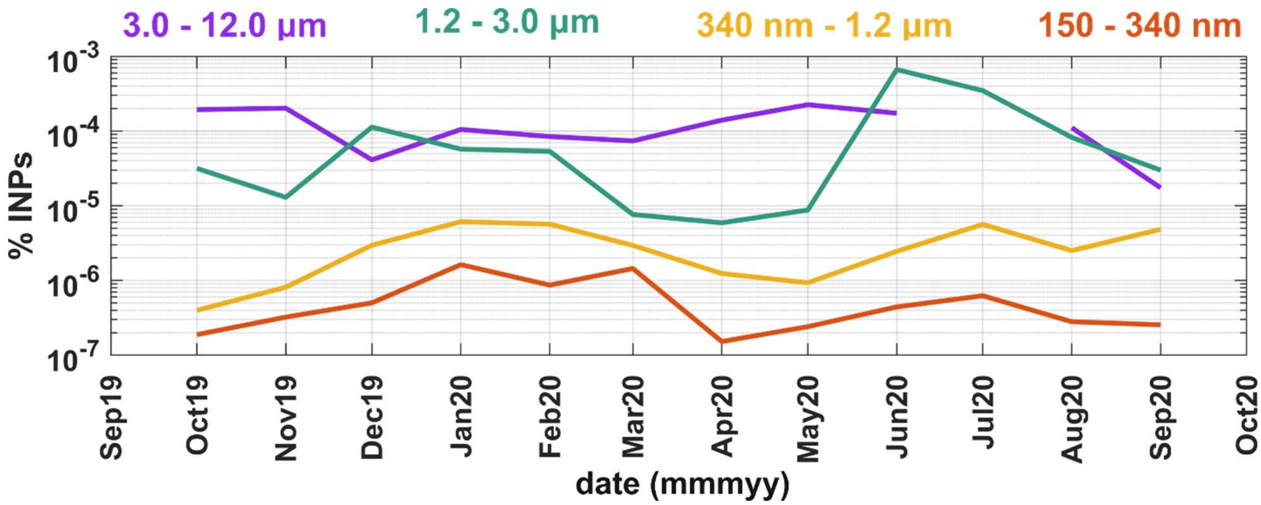

Supplementary Fig. 15. Seasonal cycle of the ratio of size-resolved total aerosol and ice nucleating particles (INPs) active at –20 °C to total aerosol, complimentary to Fig. 5 in the main text.

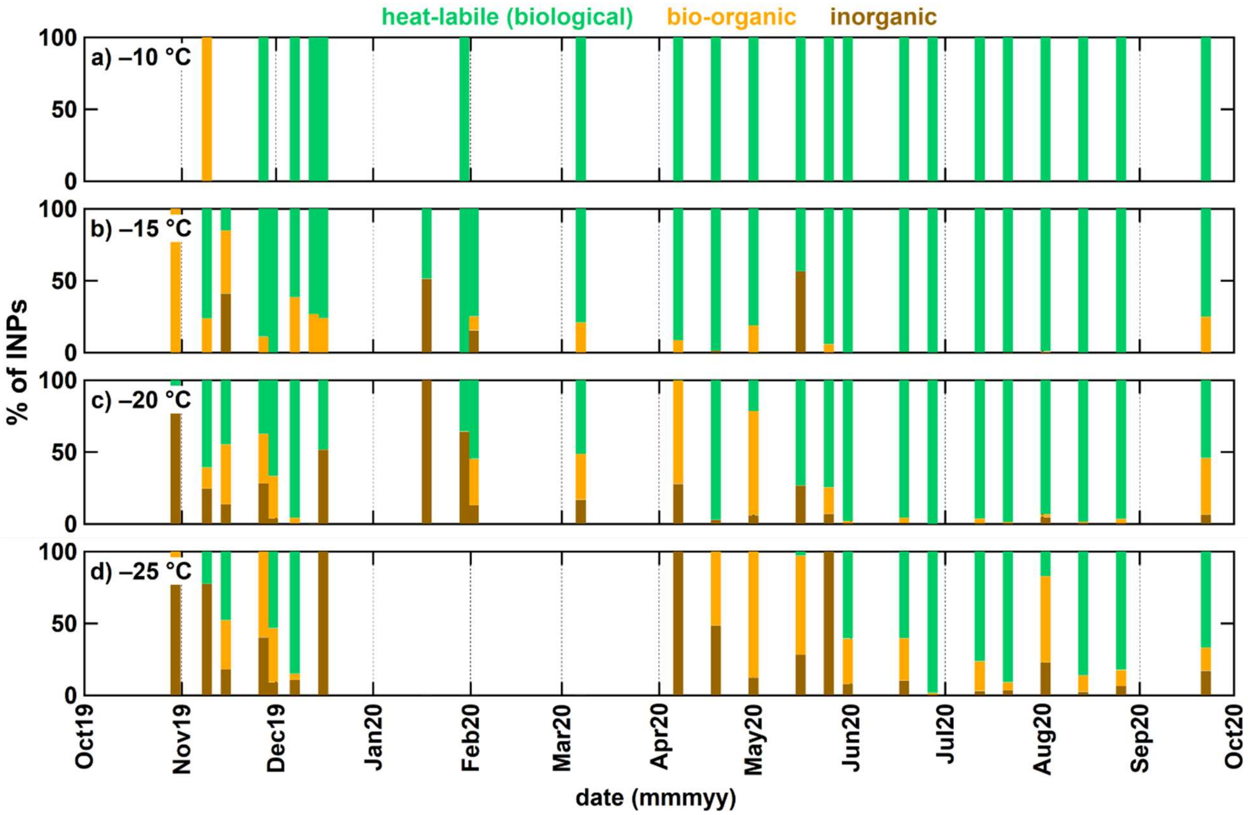

Supplementary Fig. 16. The relative abundance of heat-labile (i.e., proteinaceous or biological), bio-organic, and inorganic (i.e., mineral) ice nucleating particles (INPs) from the 72-hour total aerosol filter samples for freezing temperatures of a) –10 °C, b) –15 °C, c) –20 °C, and d) –25 °C. Select temperatures are shown to demonstrate the breadth of the varibility in INP compositon with tempreature during all seasons. Not all tempeartures contained sufficient data to determine INP type, particularly for –25 °C. Note that this method invovles filter collection of particles that overlap and occur outside the range of the Davis Rotating-drum Unit for Monitoring (DRUM) cascading impactor (i.e., smaller and larger diameters), thus, periods with little to no INPs from the DRUM at warmer temperatures may correspond to larger INP concentration here.

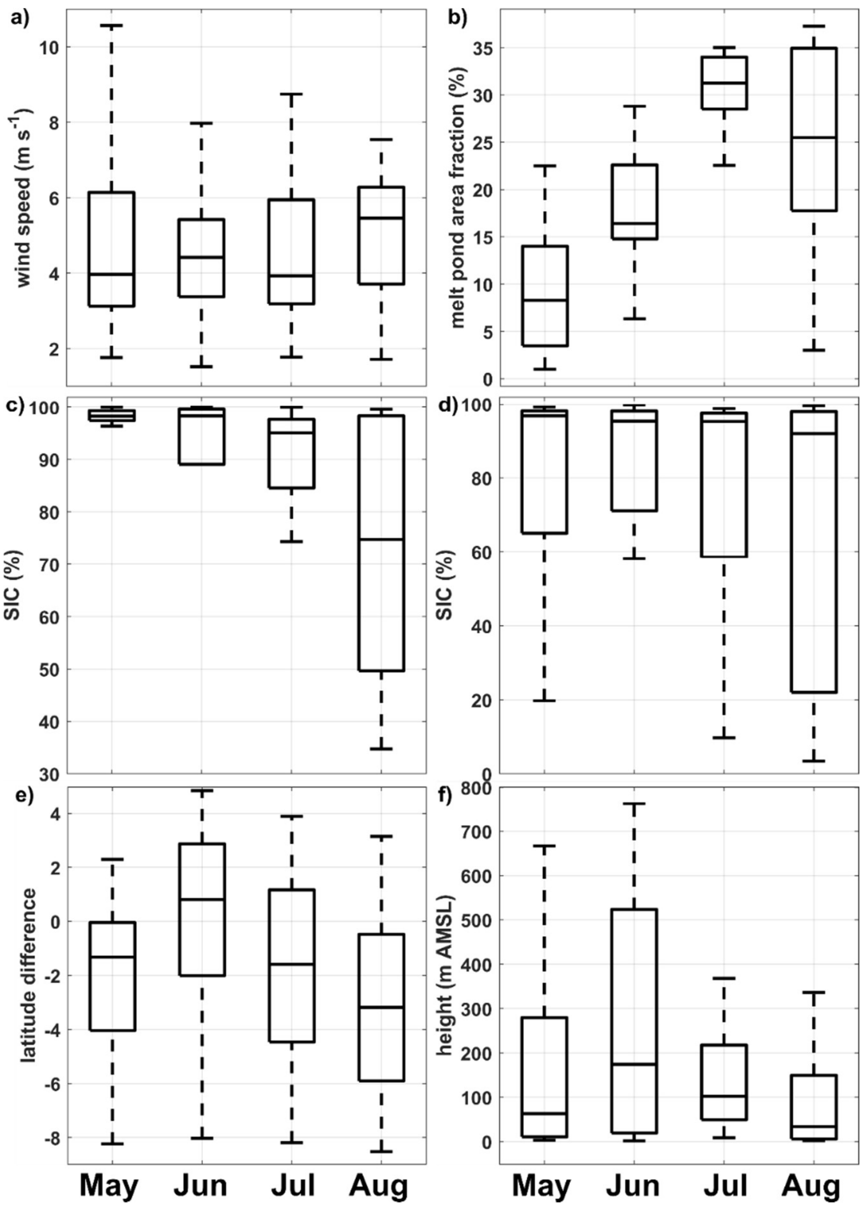

Supplementary Fig. 17. Box and whisker plots of a) wind speed, b) melt pond area fraction, c) sea ice concentration (SIC) within a 1-degree grid box around Polarstern, d) SIC along the 3-day airmass backward trajectories, e) difference in average latitude of the 3-day airmass backward trajectories and Polarstern location, and f) average height along the -day airmass backward trajectories for May through August 2020. The central mark indicates the median, and the bottom and top edges of the box indicate the 25th and 75th percentiles, respectively. The whiskers extend to the most extreme data points not considered outliers.
